# Community-Acquired *Staphylococcus aureus* Bacteremia Among People Who Inject Drugs: A National Cohort Study in England, 2017–2020

**DOI:** 10.1093/cid/ciae056

**Published:** 2024-02-05

**Authors:** Emma McGuire, Simon M Collin, Colin S Brown, Makoto Saito

**Affiliations:** Healthcare-Associated Infection (HCAI), Fungal, Antimicrobial Resistance (AMR), Antimicrobial Use (AMU), and Sepsis Division, United Kingdom Health Security Agency (UKHSA), London, UK; Department of Epidemiology and Population Health, London School of Hygiene and Tropical Medicine, London, UK; Bristol Medical School, University of Bristol, Bristol, UK; Healthcare-Associated Infection (HCAI), Fungal, Antimicrobial Resistance (AMR), Antimicrobial Use (AMU), and Sepsis Division, United Kingdom Health Security Agency (UKHSA), London, UK; National Institute for Health and Care Research Health Protection Research Unit in Healthcare Associated Infections and Antimicrobial Resistance, Imperial College London, London, UK; Division of Infectious Diseases, Advanced Clinical Research Center, Institute of Medical Science, University of Tokyo, Tokyo, Japan

**Keywords:** *Staphylococcus aureus*, people who inject drugs, bacteremia, bacterial infections, injection drug use

## Abstract

**Background:**

People who inject drugs (PWID) are at increased risk of community-acquired *Staphylococcus aureus* bacteremia (CA-SAB), but little is known about clinical outcomes of CA-SAB in PWID compared with the wider population of patients with CA-SAB.

**Methods:**

Three national datasets were linked to provide clinical and mortality data on patients hospitalized with CA-SAB in England between 1 January 2017 and 31 December 2020. PWID were identified using the *International Classification of Diseases, Tenth Revision* code for “mental health and behavioral disorder due to opioid use” (F11). Multivariable logistic regression was used to estimate adjusted odds ratios (aORs) for associations of PWID with 30-day all-cause mortality and 90-day hospital readmission.

**Results:**

In 10 045 cases of CA-SAB, 1612 (16.0%) were PWID. Overall, 796 (7.9%) patients died within 30 days of CA-SAB admission and 1189 (11.8%) patients were readmitted to hospital within 90 days of CA-SAB. In those without infective endocarditis, there was strong evidence of lower odds of mortality among PWID compared with non-PWID (aOR, 0.47 [95% confidence interval {CI}: .33–.68]; *P* < .001), whereas there was no association in CA-SAB case fatality with endocarditis (aOR, 1.40 [95% CI: .87–2.25]; *P* = .163). PWID were less likely to be readmitted within 90 days of CA-SAB (aOR, 0.79 [95% CI: .65–.95]; *P* = .011).

**Conclusions:**

In this large cohort study of patients with CA-SAB in England, PWID had lower odds of death in the absence of endocarditis and lower odds of readmission within 90 days compared to non-PWID patients. This study highlights the overrepresentation of PWID among patients with CA-SAB nationally.

## Teaser

People who inject drugs (PWID) were heavily overrepresented among patients with *Staphylococcus aureus* bacteremia nationally. PWID had lower odds of death in the absence of endocarditis and lower odds of readmission within 90 days compared to non-PWID patients.

The United Kingdom (UK) has the highest rate of drug use in Western Europe, and it is estimated that among 15- to 64-year-olds in England, 87 000 (roughly 0.2%) inject opiates and/or crack cocaine [1]. Injection drug use is associated with an increased risk of acquiring a wide range of infections including community-acquired *Staphylococcus aureus* bacteremia (CA-SAB) [2–6]. *Staphylococcus aureus* may gain entry to the bloodstream through direct inoculation via contaminated needles, through direct injection from an area of skin colonization, or through dissemination from skin infection. The opiate injectate can also be contaminated with *S. aureus* [7]. CA-SAB carries significant mortality of 10%–30% and lengthy hospitalization [8–10]. It is associated with a range of complications including severe skin and soft tissue infections (SSTIs), osteomyelitis, and infective endocarditis (IE) [11]. There are also high rates of recurrent *S. aureus* infection; the median time from completion of therapy to relapse or reinfection was 36 days (range, 10–190 days) and 99 days (range, 45–194 days), respectively [12, 13]. Approximately 22% of patients with CA-SAB are readmitted to hospital within 30 days [14].

In the UK and United States (US), a steady increase in cases of SAB linked to injection drug use was observed from 2012 until 2019 [1]. Over time, injection drug use leads to thrombosis of superficial veins; therefore, older PWID may be more likely to use sites with heavily colonized skin such as veins in the groin [3, 15]. Complex psychosocial issues such as social deprivation, homelessness, and psychiatric comorbidity, compounded by stigma associated with injection drug use and its criminalization, may adversely impact access to healthcare and lead to disproportionately poor health outcomes [16, 17].

Despite PWID making up a remarkable proportion of patients hospitalized with CA-SAB, few studies have directly compared their clinical outcomes with non-PWID patients or explored factors associated with poor clinical outcomes in this medically and socially vulnerable patient group. Available studies are limited to small single-center studies often with insufficient or no adjustment for potential confounders [16–19]. Data on clinical trajectory would be of value to clinicians and for planning public health measures to prevent CA-SAB in PWID. This study aims to determine if injection drug use is associated with poor clinical outcomes following CA-SAB in England.

## METHODS

### Data Sources and Patients

All *S. aureus* blood culture isolates with sample dates between 1 January 2017 and 31 December 2020 in England were identified through the UK Health Security Agency's Second-Generation Surveillance System (SGSS). SGSS is a voluntary laboratory surveillance system which receives reports from 98% of National Health Service (NHS) laboratories [20]. Reporting of all cases of SAB through SGSS was mandatory nationally throughout the study period. These data were linked to public NHS hospitalization data from Hospital Episode Statistics (NHS digital, London, UK) by matching deterministically on patient NHS number, date of birth, and sex [21]. Deaths were identified and linked by NHS Digital's Demographic Batch Service from the Office for National Statistics [22]. In the UK, a small minority of the population (<10%) accesses private healthcare; however, emergency presentations such as for sepsis are almost exclusively to NHS hospitals, particularly among PWID [23–25].

### Case Definition and Outcomes

CA-SAB was defined as a blood isolate taken in the period between 14 days prior and 48 hours after hospital admission that was positive for *S. aureus*. The first admission that met these criteria was considered the index admission for CA-SAB, and only the first admission was included in analyses. Transfers between hospitals within the same inpatient episode were treated as 1 admission.

The *International Classification of Diseases, Tenth Revision* (*ICD-10*) code for “mental health and behavioral disorder due to opioid use” (F11) was used as a proxy for PWID, as described previously [26, 27]. Extracted data items included age, sex, ethnicity, region, blood culture date, methicillin resistance (defined as *S. aureus* that was either methicillin or cefoxitin resistant), clinical infection syndrome, and medical, social, and psychiatric comorbidities (according to *ICD-10* codes, see Supplementary Table 1), admission and discharge dates, and date of death [28].

The primary outcome measure was 30-day all-cause mortality (from the date of index CA-SAB). All-cause readmission within 90 days of the index CA-SAB date was a secondary outcome. Patients who died within 90 days or remained in hospital >90 days were regarded as no readmission in the readmission analysis.

### Statistical Methods

All statistical analyses were performed using Stata IC 15.0 (StataCorp, College Station, Texas). Differences in clinical, microbiological, and demographic features comparing PWID with all other patients were tested using the χ^2^ test (for categorical variables) and Wilcoxon rank-sum tests (for age and length of stay [LOS]). Odds ratios (ORs) were used to assess the crude association between PWID and each outcome. Multivariable logistic regression models were built to include the following a priori confounders: age, sex, ethnicity, methicillin sensitivity, diabetes, chronic kidney disease (CKD), malignancy, psychiatric illness, homelessness, and alcohol dependence. These factors have previously been shown to have an impact on clinical outcomes (see Supplementary Figure 1 for the conceptual framework) [8, 12, 13, 29–33]. Effect modification by age, sex, homelessness, IE, and SSTI was tested by likelihood ratio test in univariable and multivariable models. Complete case analyses were conducted.

## RESULTS

### Patient Characteristics

**Figure 1.**
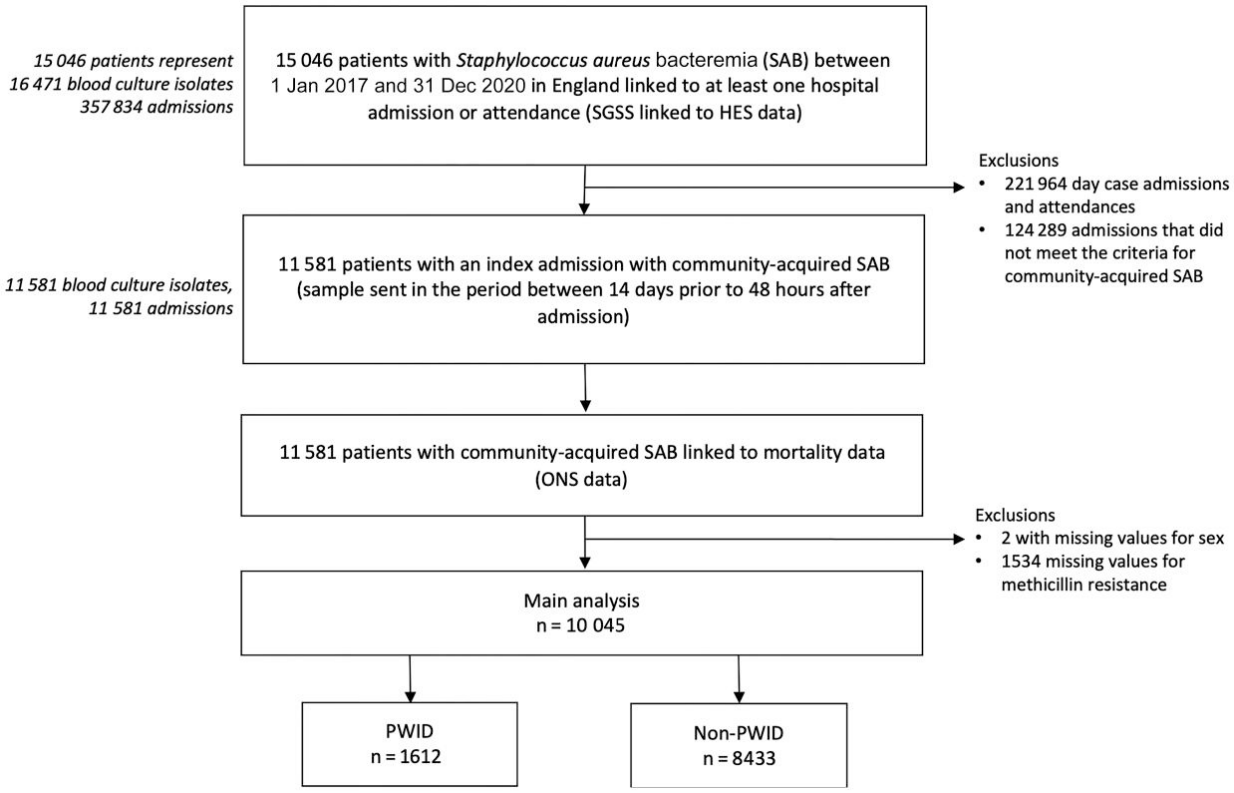
Flowchart of data linkage and exclusions for study populations of main and sensitivity analyses. Abbreviations: HES, hospital episode statistics; ONS, Office for National Statistics; PWID, people who inject drugs; SAB, *Staphylococcus aureus* bacteremia; SGSS, Second-Generation Surveillance System.

Over the 4-year study period in 2017–2020, 11 581 patients hospitalized with CA-SAB were identified. After exclusion of 1536 (13.3%) cases with missing data (2 for sex and 1534 for methicillin sensitivity), 10 045 patients were included of whom 1612 (16.0%) were PWID (Figure 1). Median age was 47 years (interquartile range [IQR], 37–55 years), and 6516 (64.9%) were male. PWID tended to be younger than non-PWID patients (median age, 40 [IQR, 34–46] years vs 46 [IQR, 38–55] years; *P* < .001). PWID were more likely to be male (73.6% vs 63.2%) and White (87.0% vs 79.0%) (Table 1). PWID had a lower prevalence of medical comorbidities (diabetes, CKD, and malignancy) compared to non-PWID patients. The prevalence of human immunodeficiency virus (HIV) observed among PWID (0.2%) was lower than among non-PWID (1.2%). However, PWID had a higher prevalence of methicillin-resistant *S. aureus* (MRSA) (8.2% vs 6.7%, *P* = .020) and were more likely to have psychiatric comorbidity (25.2% vs 17.8%, *P* < .001) and to experience homelessness (10.2% vs 1.1%, *P* < .001). PWID were also more likely to have IE (19.5% vs 6.6%, *P* < .001), pneumonia (22.0% vs 14.4%, *P* < .001), or SSTI (39.2% vs 19.9%, *P* < .001).

**Table 1.** Demographics, Methicillin Resistance, Comorbidities and Associated Clinical Infection Syndromes by Opiate Dependence Status in Patients Hospitalized With Community-Acquired *Staphylococcus aureus* Bacteremia in England, 1 January 2017 to 31 December 2020 (N = 10 045)

Characteristic	Non-PWID (n = 8433), No. (%)	PWID (n = 1612), No. (%)	*P* Value^a^
Demographics			
Age, y			<.001
18–30	993 (11.8)	196 (12.2)	
31–40	1486 (17.6)	672 (41.7)	
41–50	2119 (25.1)	570 (35.4)	
51–60	3835 (45.5)	174 (10.8)	
Sex			<.001
Male	5329 (63.2)	1187 (73.6)	
Female	3104 (36.8)	425 (26.4)	
Ethnicity			<.001
White	6659 (79.0)	1402 (87.0)	
Asian, Black, Caribbean, or African	455 (5.4)	13 (0.8)	
Mixed, unknown, or other	1319 (15.6)	197 (12.2)	
Region			<.001
London	1045 (12.4)	125 (7.8)	
East Midlands	704 (8.4)	130 (8.1)	
East of England	852 (10.1)	107 (6.6)	
North-East	564 (6.7)	125 (7.8)	
North-West	1284 (15.2)	274 (17.0)	
South-East	1169 (13.9)	139 (8.6)	
South-West	987 (11.7)	234 (14.5)	
West Midlands	974 (11.6)	186 (11.5)	
Yorkshire and The Humber	854 (10.1)	292 (18.1)	
Methicillin resistance			
MRSA	556 (6.7)	132 (8.2)	.020
Comorbidities			
Diabetes	2112 (25.0)	49 (3.0)	<.001
HIV infection	99 (1.2)	3 (0.2)	<.001
CKD stage 3–5	831 (9.9)	18 (1.1)	<.001
Malignancy	932 (11.0)	9 (0.6)	<.001
Alcohol dependence	839 (10.0)	159 (9.9)	.916
Psychiatric comorbidity	1502 (17.8)	406 (25.2)	<.001
Homelessness	92 (1.1)	165 (10.2)	<.001
Clinical infection syndrome			
Infective endocarditis	552 (6.6)	315 (19.5)	<.001
Pneumonia	1216 (14.4)	355 (22.0)	<.001
Bone or joint infection	420 (5.0)	84 (5.2)	.698
Skin or soft tissue infection	1679 (19.9)	632 (39.2)	<.001
Device infection	966 (11.4)	31 (1.1)	<.001

Cases with missing values for sex (n = 2) and MRSA (n = 1534) have been excluded.

Abbreviations: HIV, human immunodeficiency virus; CKD, chronic kidney disease; MRSA, methicillin-resistant *Staphylococcus aureus*; PWID, people who inject drugs.

^a^ *P* values derived from χ^2^ tests.

### Mortality

Death from any cause within 30 days of CA-SAB admission occurred in 796 of 10 045 (7.9%) total patients and in 76 of 1612 (4.7%) and 720 of 8433 (8.5%) PWID and non-PWID, respectively (Table 2). PWID had half the crude odds of 30-day mortality when compared to non-PWID patients (OR, 0.53 [95% confidence interval {CI}: .42–.68]; *P* < .001). In a multivariable model there was no evidence for an association between PWID and 30-day all-cause mortality (adjusted OR [aOR], 0.79 [95% CI: .61–1.03]; *P* = .083). However, there was evidence for effect modification by the presence of IE (*P* < .001). A strong protective effect between PWID and mortality was observed in patients without IE (aOR, 0.47 [95% CI: .33–.68]; *P* < .001), whereas there was no association in patients with IE (aOR, 1.40 [95% CI: .87–2.25]; *P* = .163) (Table 3). There was no other significant effect modification (Supplementary Table 3).

**Table 2.** Frequency of 30-Day Mortality Among Injection Drug Use and Other Covariate Groups, and Unadjusted Odds Ratios (95% Confidence Intervals) for Their Association With 30-Day All-Cause Mortality (N = 10 045)

Characteristic	Died Within 30 d (n = 796), No. (%)	Unadjusted OR^a^ (95% CI)	*P* Value^b^
Injection drug use			<.001
Non-PWID	720 (8.5)	…	
PWID	76 (4.7)	0.53 (.42–.68)	
Demographics			
Age, y		…	<.001
18–30	27 (2.3)	…	
31–40	101 (4.7)	2.11 (1.37–3.25)	
41–50	217 (8.1)	3.78 (2.51–5.69)	
51–60	451 (11.2)	5.46 (3.68–8.09)	
Sex			.736
Male	512 (7.9)	…	
Female	284 (8.1)	1.03 (.88–1.19)	
Ethnicity			.235
White	652 (8.1)	…	
Asian, Black, Caribbean, or African	40 (8.5)	1.06 (.76–1.48)	
Mixed, unknown, or other	104 (6.9)	0.84 (.68–1.04)	
Region			.237
London	69 (5.9)	…	
East Midlands	70 (8.4)	1.46 (1.04–2.06)	
East of England	81 (8.4)	1.47 (1.06–2.05)	
North-East	53 (7.7)	1.33 (.92–1.93)	
North-West	134 (8.6)	1.50 (1.11–2.03)	
South-East	104 (7.9)	1.38 (1.01–1.89)	
South-West	96 (7.9)	1.36 (.99–1.88)	
West Midlands	86 (7.4)	1.28 (.92–1.77)	
Yorkshire and The Humber	103 (9.0)	1.58 (1.15–2.16)	
Methicillin resistance			
MSSA	731 (7.8)	…	.125
MRSA	65 (9.4)	1.23 (.94–1.61)	
Comorbidities			
No diabetes	621 (7.9)	…	.736
Diabetes	175 (8.1)	1.03 (.87–1.23)	
No HIV infection	779 (7.8)	…	.001
HIV infection	17 (16.7)	2.35 (1.39–3.98)	
No chronic kidney disease	727 (7.9)	…	.819
Chronic kidney disease	69 (8.1)	1.03 (.80–1.33)	
No malignancy	622 (6.8)	…	<.001
Malignancy	174 (19.5)	3.09 (2.57–3.72)	
No alcohol dependence	659 (7.3)	…	<.001
Alcohol dependence	137 (13.7)	2.03 (1.66–2.47)	
No psychiatric comorbidity	685 (8.4)	…	<.001
Psychiatric comorbidity	111 (5.8)	0.67 (.55–.83)	
No homelessness	785 (8.0)	…	.028
Homelessness	11 (4.3)	0.51 (.28–.94)	
Clinical infection syndrome			
No infective endocarditis	689 (7.5)	…	<.001
Infective endocarditis	107 (12.3)	1.73 (1.40–2.15)	
No pneumonia	585 (6.9)	…	<.001
Pneumonia	211 (13.4)	2.09 (1.77–2.47)	
No bone or joint infection	783 (8.2)	…	<.001
Bone or joint infection	13 (2.6)	0.30 (.17–.52)	
No skin or soft tissue infection	694 (9.0)	…	<.001
Skin or soft tissue infection	102 (4.4)	0.47 (.38–.58)	
No device infection	762 (8.4)	…	<.001
Device infection	34 (3.4)	0.38 (.27–.54)	

Cases with missing values for sex (n = 2) and MRSA status (n = 1534) have been excluded.

Abbreviations: CI, confidence interval; HIV, human immunodeficiency virus; MRSA, methicillin-resistant *Staphylococcus aureus*; MSSA, methicillin-sensitive *Staphylococcus aureus*; OR, odds ratio; PWID, people who inject drugs.

^a^Unadjusted ORs estimated using logistic regression.

^b^ *P* values derived from χ^2^ tests.

**Table 3.** Outcomes of People Who Inject Drugs (PWID) With Community-Acquired *Staphylococcus aureus* Bacteremia (CA-SAB) Compared to Non-PWID Patients With CA-SAB in England (N = 10 045)

Outcome	Non-PWID	PWID	Unadjusted OR^a^ (95% CI)	Adjusted OR^a^ (95% CI)	*P* Value^b^
30-d all-cause mortality	720/8433	76/1612	0.53 (.42–.68)	0.79 (.61–1.03)	.083
By IE strata					
No IE	654/7881	35/1297	…	0.47 (.33–.68)	<.001
IE	66/552	41/315	…	1.40 (.87–2.25)	.163
90-d all cause readmission	1019/8433	170/1612	0.86 (.72–1.02)	0.79 (.65–.95)	.011

Cases with missing values for sex (n = 2) and methicillin-resistant *Staphylococcus aureus* (n = 1534) have been excluded.

Abbreviations: CI, confidence interval; IE, infective endocarditis; OR, odds ratio; PWID, people who inject drugs.

^a^Crude and adjusted ORs estimated using logistic regression; overall and stratum-specific *P* values derived from the Wald test.

^b^Adjusted by age, sex, ethnicity, methicillin sensitivity, diabetes, chronic kidney disease, malignancy, psychiatric illness, homelessness, and alcohol dependence (human immunodeficiency virus excluded due to data sparsity).

### Readmission

Overall, 1189 of 10 045 (11.8%) patients were readmitted to hospital within 90 days of CA-SAB, comprising 170 of 1612 (10.5%) and 1019 of 8433 (12.1%) among PWID and non-PWID patients, respectively (Supplementary Table 2). There was weak evidence that PWID had lower crude odds of 90-day readmission when compared to non-PWID patients (unadjusted OR, 0.86 [95% CI: .72–1.02]; *P* = .080). In a multivariable logistic regression model, there was evidence for a protective effect of PWID on the odds of readmission to hospital within 90 days of index CA-SAB (aOR, 0.79 [95% CI: .65–.95]; *P* = .011) (Table 3).

### Length of Stay

For the CA-SAB cohort overall, the median LOS was 13 days (IQR, 5–24 days). PWID tended to have a longer duration of hospital stay than non-PWID patients, with a median of 15 days (IQR, 5–27 days) and 13 days (IQR, 5–23 days), respectively (*P* < .001).

## DISCUSSION

People who inject drugs were heavily overrepresented in this large national patient cohort representing 16% of hospitalized patients with CA-SAB compared to 0.2% in the general population [1]. CA-SAB is a common infection with associated remarkable morbidity and mortality, but little is known about clinical outcomes in this marginalized patient group. Our data show that PWID had lower odds of death in the absence of endocarditis and were less likely to be readmitted to hospital within 90 days compared to non-PWID patients. This lower mortality was possibly because bacteremia in PWID largely relates to specific behavior rather than underlying illness. The age of PWID in this study was younger than non-PWID patients, consistent with a median age of 41 years (IQR, 36–48 years) for the PWID population in England [6, 34]. This may account for the lower prevalence of medical comorbidities observed among PWID, as has been reported previously [19]. Diabetes, HIV, CKD, and malignancy have been associated with increased risk of recurrent infection and death following SAB [12, 13, 33]. In our cohort overall, only HIV and malignancy were significantly associated with increased unadjusted odds of death, and diabetes and CKD were associated with lower odds of readmission.

PWID may also be more prone to transient bacteremia or bacteremia due to less virulent strains of *S. aureus*. Direct inoculation of *S. aureus* into the bloodstream bypasses primary defence mechanisms including the skin, immune cells in the subcutaneous tissues, and the blood vessel wall. This may allow relatively poorly pathogenic strains of *S. aureus* (lacking in virulence factors) to produce a bacteremia, with milder associated clinical syndromes. This issue has recently been investigated in a case-control study conducted in Missouri, US, by Marks et al [35]. The authors found that PWID had lower 1-year mortality associated with SAB (14.3% vs 29.7%). Phylogenetic and comparative genomic analyses demonstrated that intravenous drug use–associated bloodstream infections (IDU-BSIs) grouped into multiple unique clonal clusters and that *S. aureus* strains lacking in classical virulence factors were overrepresented among IDU-BSIs. Despite the lower mortality observed, they also described considerable complications and morbidity associated with CA-SAB in PWID including prolonged bacteremia and higher prevalence of IE [18].

Only 1 previous study made direct comparisons of clinical outcomes in PWID and non-PWID patients with CA-SAB. This was a single-center study by McClellan et al of 248 patients in Oregon, US [19]. The authors reported a weak trend toward lower risk of 90-day mortality (4.3% vs 9.6%, *P* = .170) and increased risk of readmission within 90 days (46.3% vs 39.8%, *P* = .154) among PWID compared to non-PWID patients. While PWID were more likely to have MRSA bacteremia in both studies, the prevalence of MRSA in SAB in the US (48.5%) is much higher than in the UK (6.8% in this study) [14, 36]. Methicillin resistance has been associated with increased LOS, readmission, and death in SAB [13, 14]. However, while patients with MRSA in our study had increased LOS compared to those with methicillin-sensitive *S. aureus*, there was no association with mortality, and they had lower odds of readmission.

One study based in London noted that PWID had frequent hospital admissions (up to 12 over a 4-year period) [18]. PWID have also been reported to have high rates of discharge from hospital against medical advice—25.7% in PWID versus 1.1% in non-PWID in a US study and 15% in PWID in a UK study [16, 19]. However, we observed a lower risk of readmission among PWID possibly because LOS was slightly longer for PWID as was reported by McClellan et al [19]. In the UK, CA-SAB is generally treated in hospital with intravenous (IV) antibiotics for a minimum of 2 weeks, after which patients requiring longer courses can complete IV therapy via outpatient parenteral antibiotic therapy (OPAT) [29]. However, OPAT is often not considered possible for PWID due to challenges in delivering IV therapy [10, 16]. Clinicians may have reservations about discharging patients who inject drugs with venous access in situ due to concern that the cannula could be used to inject drugs. This, along with the higher prevalence of psychosocial issues experienced by PWID, may contribute to delays in hospital discharge.

A high burden of homelessness was observed within the PWID population (10.2% vs 1.1% in non-PWID), in keeping with findings from the Washington-based study by Marks et al (12% vs 0%) [35]. However, even this figure is likely to be an underestimate. A survey of PWID in England in 2008 found that 86% had been homeless at some stage and 59% over the past month [6]. PWID may have different experience of homelessness, such as a higher rate of street homelessness, which might differentially affect clinical outcomes in SAB [2–4, 32]. In our overall cohort, homelessness was associated with lower odds of death but was not associated with readmission. This unexpected finding may relate to the younger age of the homeless population (only 12% of homeless people are aged >55 years); however, in 2021 the average age of death of men and women experiencing homelessness was 45 and 43 years, respectively [37]. However, this finding may simply reflect the broad definition of homelessness that we used or misclassification due to inaccurate coding.

The prevalence of HIV observed among PWID (0.2%) was unexpectedly lower than among non-PWID (1.2%) and lower than estimates of the prevalence of HIV among PWID in England, which range from 0.8% to 1.1% [1, 6]. Our study relied on *ICD-10* coding for classification of HIV status, and low uptake of screening may have led to an underestimate of the burden of HIV in PWID. In 2021, 18% of PWID who were in contact with services and currently inject reported never having had an HIV test [1]. Disruptions to HIV testing during the pandemic may have contributed to poorer screening during the study period. In 2021, immediately after our study period, the UK government's Zero HIV action plan recommended switching to an opt-out HIV testing strategy in areas with the highest HIV prevalence [38]. Many acute urban centers now offer opt-out HIV testing, so the HIV prevalence in this study may not be reflective of the current picture.

PWID were more likely to have endocarditis, SSTI, and pneumonia, consistent with single-center studies [18, 19]. For example, McClellan et al reported that, among hospitalized patients with SAB, PWID had higher rates of IE (31.4% vs 8.5%, *P* < .001) and of SSTI (25.7% vs 14.8%, *P* = .02) than non-PWID [19]. In patients with SAB, endocarditis and pneumonia are associated with increased risk of SAB recurrence, readmission, mortality, and increased LOS [8, 9, 12, 33]. The higher prevalence of both conditions observed among PWID may reflect delays in presentation, suggesting outcomes could be improved with better access to healthcare [6, 39–41]. PWID may also be affected by a different spectrum of SSTIs (eg, groin abscess and chronic fistulae) which may affect clinical outcome [6, 15, 42].

A considerable strength of our study is that the linked data from 3 national datasets we used offer national coverage inclusive of a broad demographic, enhancing the generalizability of our findings. Use of national hospitalization and mortality datasets enabled identification of readmissions and deaths across different NHS Trusts or geographic regions, which is particularly relevant because PWID may be a more mobile population due to higher risk of homelessness. Potential confounding by comorbidities known to be risk factors for mortality and readmission in SAB was mitigated by adjusting for these factors.

A limitation of our study is that the national datasets lack information on individual patient management, such as antibiotic regimens and infection specialist review. These treatment factors are known to influence outcomes in SAB, and it is possible that they differ between PWID and non-PWID patients [8, 9, 11, 29–31, 43]. Clinicians may, for example, opt for oral antibiotics for PWID due to difficulties with IV access [16]. Poorer clinical outcomes due to these alternative regimens, however, would act in the opposite direction to our finding of lower 30-day mortality. Higher mortality rates can lead to apparently lower readmission rates (ie, competing risks), but both mortality and readmission rates were lower in PWID in our study. Potential unmeasured confounders include smoking, socioeconomic status, and differences between urban and rural settings. We also did not have access to data on whether endocarditis was left-sided or right-sided. This represents another source of potential confounding because the latter is known to be particularly associated with injection drug use and has better clinical outcomes.

The F11 *ICD-10* code was used as a proxy to identify PWID. In England, the use of *ICD-10* codes for mental health disorders has been validated with reasonable certainty [44]. Additionally, according to the National Statistics in England, only minority (circa 2%) of nonopiate users inject drugs in 2021 [34]. When used in conjunction with inclusion criteria of admission due to SAB, this code is likely to be more specific for individuals who are currently injecting. The proportion of CA-SAB cases designated as PWID in our study is comparable to previous reports of 10%–20% in England over this period [1].

The final year of our 4-year study period included the first 9 months of the coronavirus disease 2019 (COVID-19) pandemic in England. Public health measures may have impacted PWID, potentially limiting access to safe injecting equipment and blood-borne virus testing [1]. However, the COVID-19 pandemic represented only 9 months of the 48-month study period and is unlikely to have had a significant effect on our overall findings.

Our study highlights the overrepresentation of PWID among patients with CA-SAB and provides information that could be helpful for health services and public health intervention planning. A proportion of CA-SAB is likely to be preventable through public health actions targeted at PWID, including education regarding risks of bacterial infection and safe injecting practices, improved access to opiate substitution programs, and provision of clean needles and wound care packs [1–3, 6, 27, 34]. Prospective observational studies with data on injecting behavior and detailed care pathway and treatment data could inform the design of complex intervention studies aimed at reducing the burden of infection experienced by PWID patients by addressing the myriad of health and psychosocial factors they face. Further studies delineating *S. aureus* lineages and virulence factors in the PWID population in the UK as well as preclinical studies exploring lineage-dependent pathogenesis would be of value.

## Supplementary Data

Supplementary materials are available at *Clinical Infectious Diseases* online. Consisting of data provided by the authors to benefit the reader, the posted materials are not copyedited and are the sole responsibility of the authors, so questions or comments should be addressed to the corresponding author.

## Supplementary Material

ciae056_Supplementary_Data

## Notes

***Author Contributions.*** E. M.: Conceptualization, formal analysis, methodology, data interpretation, writing. S. M. C.: Methodology, data interpretation, and supervision. C. S. B.: Methodology, data interpretation, and supervision. M. S.: Methodology, data interpretation, and supervision. All authors reviewed results, provided guidance on methods, revised the manuscript, and approved the final manuscript.

***Acknowledgments.*** The views expressed in this article are those of the authors and not necessarily those of the National Health Service (NHS), the National Institute for Health and Care Research Imperial Biomedical Research Centre, the University of Tokyo, or the UK Health Security Agency.

***Data availability.*** The Stata files (containing the commands used) can be made available on request. However, the de-identified individual patient data utilized in this study are the property of NHS Digital and/or UK Health Security Agency and cannot be made available. Please contact the corresponding author for further information.

***Financial support*.** This research received no specific grant from any funding agency in the public, commercial, or not-for-profit sectors.
