# Supplementary material for: Community-Acquired Staphylococcus aureus Bacteremia Among People Who Inject Drugs: A National Cohort Study in England, 2017–2020
Source: Clin Infect Dis. 2024 Feb 5;78(6):1443–50. doi: 10.1093/cid/ciae056 (PMC11175704; doi:10.1093/cid/ciae056)
Supplement: ciae056_Supplementary_Data [file ciae056_supplementary_data.docx]

# Supplementary material for “Community-acquired Staphylococcus aureus bacteraemia among people who inject drugs: a national cohort study in England, 2017-2020”

Contents

[Supplementary material for “Community-acquired Staphylococcus aureus bacteraemia among people who inject drugs: a national cohort study in England, 2017-2020” 1](#_Toc154750077)

[**Supplementary Figure S1.** Conceptual framework for the association between injection drug use and clinical outcomes in patients admitted to hospital with community-acquired *Staphylococcus aureus* bacteraemia. 2](#_Toc154750078)

[**Supplementary Table S1.** Summary of International Classification of Disease (ICD-10) codes used to identify injection drug use status, clinical infection syndromes, and medical, social and psychiatric co-morbidities. 3](#_Toc154750079)

[**Supplementary Table S2.** Frequency of 90-day readmission among injecting drug use & other covariate groups, and unadjusted odds ratios (95% confidence intervals) for their association with 90-day hospital readmission (n=10,045). 4](#_Toc154750080)

[**Supplementary Table S3.** Stratification of odds ratios (95% confidence intervals) and tests for interaction for potential effect modifiers of the association between injection drug use and each clinical outcome (n=10,045). 6](#_Toc154750081)

## **Supplementary Figure S1.** Conceptual framework for the association between injection drug use and clinical outcomes in patients admitted to hospital with community-acquired *Staphylococcus aureus* bacteraemia.


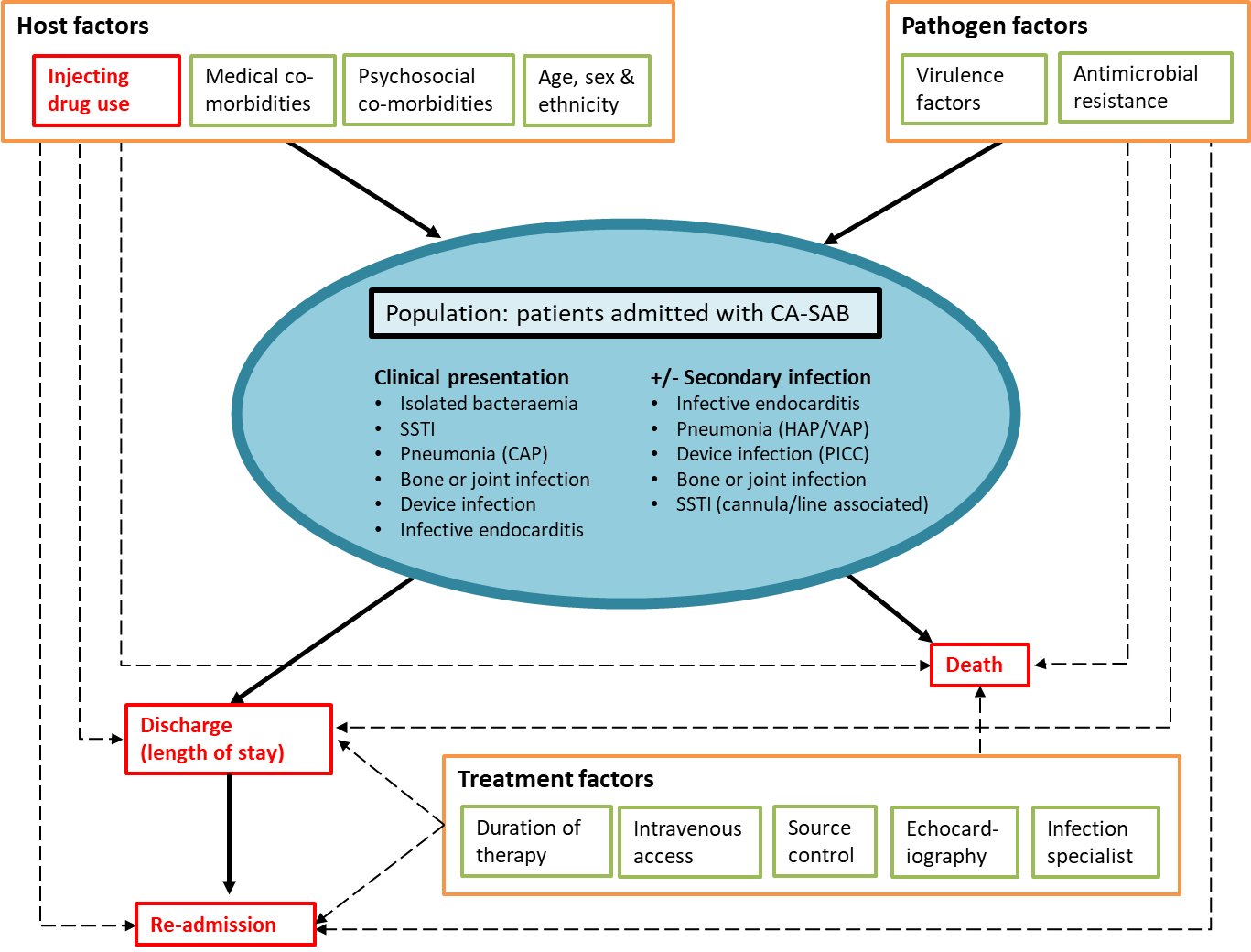


CA-SAB: community-acquired *Staphylococcus aureus;* SSTI: skin or soft tissue infection; CAP: community-acquired pneumonia; HAP: hospital acquired pneumonia; VAP: ventilator-associated pneumonia; PICC: peripherally inserted central catheter

## **Supplementary Table S1.** Summary of International Classification of Disease (ICD-10) codes used to identify injection drug use status, clinical infection syndromes, and medical, social and psychiatric co-morbidities.

| **Category** | **Icd10 codes** |
| --- | --- |
| Mental health and behavioural disorder due to opioid use (used for this study as a surrogate for injection drug use) | F111-F119 |
| Infective endocarditis | I330*I339*I38X*T826 |
| Pneumonia | J152*J158*J159*J180*J181*J188*J189*J850*J851*J852*J853*J860**J869*P232*P239 |
| Skin and/or soft tissue infection | A46*A480*H01*J430*K122*K610*K611*K612*K613*K614*L00*L080*L081*L088*L089*L010*L020*L021*L022*L023*L024*L028*L029*L030*L031*L032*L033*L038*L039*L040*L050*L059*M600*M609*O860*O911*P394*R02*T874 |
| Bone and/or joint infection | M000*M009*T845*T846 |
| Infection of prosthetic devices, implants and grafts | T827*T825*T836*T857 |
| Diabetes mellitus | E10*E11*E13*E14 |
| Human immunodeficiency virus infection | B20*B21*B22*B23*B24*O42*097*O98*B97*V08*Z21 |
| Chronic kidney disease stages 3-5 | N183*N184*N185 |
| Malignancy (solid tumour or malignancy of the haematopoietic or lymphoid tissue) | C15*C16*C17*C18*C19*C20*C21*C22*C23*C24*C25*C26*C43*C44*C50*C51*C52*C53*C54*C55*C56*C56*C57*C58*C73*C74*C75*C76*C77*C78*C79*C80*C81*C82*C83*C84*C85*C86*C87*C88*C89*C90*C91*C92*C93*C94*C95*C96*D00*D01*D02*D03*D04*D05*D06*D07*D08*D09 |
| Homelessness | Z590 |
| Alcohol dependence | F100-F109 |
| Psychiatric co-morbidity | F200/F209 (schizophrenia), F300/F390 (depression or bipolar disorder), F400/F419 (anxiety) |

## **Supplementary Table S2.** Frequency of 90-day readmission among injecting drug use & other covariate groups, and unadjusted odds ratios (95% confidence intervals) for their association with 90-day hospital readmission (n=10,045).

| **Characteristic** | **Readmitted within 90 days**  **(N=1189)**  **n (%)** | **Unadjusted OR***  **(95% CI)** | **P value**** |
| --- | --- | --- | --- |
| Injecting drug use  Non-PWID  PWID | 1019 (12.1%)  170 (10.5%) | --  0.86 (0.72-1.02) | 0.080 |
| Age  18-30 years  31-40 years  41-50 years  51-60 years | 153 (12.9%)  225 (10.4%)  325 (12.1%)  486 (12.1%) | --  0.79 (0.63-0.98)  0.93 (0.76-1.14)  0.93 (0.77-1.13) | 0.122 |
| Sex  Male  Female | 792 (12.1%)  397 (11.2%) | --  0.92 (0.81-1.04) | 0.180 |
| Ethnicity  White  Asian, Black, Caribbean or African  Mixed, unknown or other | 964 (12.0%)  47 (10.0%)  178 (11.7%) | --  0.82 (0.60-1.11)  0.98 (0.83-1.16) | 0.456 |
| Geographic Region  London  East Midlands  East of England  North East  North West  South East  South West  West Midlands  Yorkshire and The Humber | 134 (11.4%)  105 (12.6%)  135 (14.1%)  98 (14.2%)  175 (11.2%)  162 (12.4%)  129 (10.6%)  138 (11.9%)  113 (9.9%) | --  1.11 (0.85-1.46)  1.27 (0.98-1.64)  1.28 (0.97-1.70)  0.98 (0.77-1.24)  1.09 (0.86-1.39)  0.91 (0.71-1.18)  1.04 (0.81-1.34)  0.85 (0.65-1.10) | 0.041 |
| Methicillin sensitivity  MSSA  MRSA | 1128 (12.1%)  61 (9.9%) | --  0.71 (0.54-0.93) | 0.012 |
| Diabetes  No diabetes  Diabetes | 990 (12.6%)  199 (9.2%) | --  0.71 (0.60-0.83) | <0.001 |
| HIV infection  No HIV  HIV | 1175 (11.8%)  14 (13.7%) | --  1.19 (0.67-2.09) | 0.553 |
| Chronic kidney disease (CKD)  No CKD  CKD | 1134 (12.3%)  55 (6.5%) | --  0.49 (0.37-0.65) | <0.001 |
| Malignancy  No malignancy  Malignancy | 1075 (11.8%)  114 (12.1%) | --  1.03 (0.84-1.26) | 0.782 |
| Alcohol dependence  No alcohol dependence  Alcohol dependence | 1076 (11.9%)  113 (11.3%) | --  0.95 (0.77-1.16) | 0.596 |
| Psychiatric co-morbidity  No psychiatric co-morbidity  Psychiatric co-morbidity | 971 (11.9%)  218 (11.4%) | --  0.95 (0.81-1.11) | 0.537 |
| Homelessness  No homelessness  Homelessness | 1158 (11.8%)  31 (12.1%) | --  1.02 (0.70-1.50) | 0.910 |
| Infective endocarditis (IE)  No IE  IE | 1078 (11.7%)  111 (12.8%) | --  1.10 (0.86-1.36) | 0.357 |
| Pneumonia  No pneumonia  Pneumonia | 1016 (12.0%)  173 (11.0%) | --  0.91 (0.77-1.08) | 0.271 |
| Bone or joint infection (BJI)  No BJI  BJI | 1140 (11.9%)  49 (9.7%) | --  0.79 (0.59-1.07) | 0.132 |
| Skin or soft tissue infection (SSTI)  No SSTI  SSTI | 922 (11.9%)  267 (11.5%) | --  0.97 (0.83-1.12) | 0.631 |
| Device infection  No device infection  Device infection | 1101 (12.2%)  88 (8.8%) | --  0.70 (0.56-0.88) | 0.002 |

OR: odds ratio, CI: confidence interval, PWID: people who inject drugs; MRSA: methicillin resistant *Staphylococcus aureus*, MSSA: methicillin sensitive *Staphylococcus aureus*, HIV: human immunodeficiency virus; CKD: chronic kidney disease

*Unadjusted ORs estimated using logistic regression

**p values derived from X^2^ tests

NB: cases with missing values for sex (n=2) and MRSA status (n=1,534) have been excluded

### **Supplementary Table S3.** Stratification of odds ratios (95% confidence intervals) and tests for interaction for potential effect modifiers of the association between injection drug use and each clinical outcome (n=10,045).

|  | **30-day mortality** | | **90-day hospital re-admission** | |
| --- | --- | --- | --- | --- |
| **Characteristic** | **Stratified OR**  **(95% CI)** | **P value*** | **Stratified OR**  **(95% CI)** | **P value*** |
| Age  18-30 years  31-40 years  41-50 years  51-60 years | 0.88 (0.30-2.57)  0.72 (0.45-1.14)  0.62 (0.42-0.92)  0.74 (0.42-1.26) | 0.909 | 0.88 (0.55-1.42)  0.89 (0.65-1.20)  0.88 (0.66-1.18)  0.88 (0.54-1.44) | 1.000 |
| Sex  Male  Female | 0.48 (0.36-0.65)  0.65 (0.43-1.01) | 0.267 | 0.81 (0.66-1.00)  0.95 (0.69-1.32) | 0.427 |
| Homelessness  No homelessness  Homelessness | 0.53 (0.41-0.69)  0.97 (0.28-3.42) | 0.348 | 0.85 (0.71-1.02)  0.87 (0.40-1.88) | 0.956 |
| Infective endocarditis  No infective endocarditis  Infective endocarditis | 0.31 (0.22-0.43)  1.10 (0.73-1.67) | <0.001 | 0.83 (0.69-1.01)  0.90 (0.59-1.37) | 0.733 |
| Skin or soft tissue infection  No skin or soft tissue infection  Skin or soft tissue infection | 0.61 (0.46-0.80)  0.56 (0.33-0.93) | 0.780 | 0.82 (0.66-1.02)  0.94 (0.70-1.25) | 0.473 |

OR: odds ratio; CI: confidence interval.

*p values derived from likelihood ratio test for interaction.

NB: cases with missing values for sex (n=2) and methicillin resistance (n=1,534) have been excluded.
